# High prevalence of secondary factors for bone fragility in patients with a recent fracture independently of BMD

**DOI:** 10.1007/s11657-016-0258-3

**Published:** 2016-02-23

**Authors:** F. Malgo, N. M. Appelman-Dijkstra, M. F. Termaat, H. J. L. van der Heide, I. B. Schipper, T. J. Rabelink, N. A. T. Hamdy

**Affiliations:** 1 Center for Bone Quality and Department of Medicine, Leiden University Medical Center, P.O. Box 9600, 2300 RC Leiden, The Netherlands; 2 Center for Bone Quality and Department of Traumatology, Leiden University Medical Center, Leiden, The Netherlands; 3 Center for Bone Quality and Department of Orthopaedic Surgery, Leiden University Medical Center, Leiden, The Netherlands

**Keywords:** Secondary factors, Fracture liaison service, Fragility fracture, Osteopenia, Osteoporosis

## Abstract

***Summary*:**

In this study, we demonstrate a high prevalence of secondary factors in patients with a recent fracture independently of bone mineral density (BMD). Our results suggest that patients with a recent fracture should be screened for secondary factors for bone fragility regardless of BMD values.

**Introduction:**

Secondary factors for bone fragility are common in patients with osteoporosis who have sustained a fracture. The majority of fragility fractures occurs, however, in patients with osteopenia, and it is not known whether secondary factors may contribute to fracture risk in these patients or in those with normal BMD.

**Methods:**

Prospective cohort study evaluating the prevalence of secondary factors for bone fragility in consecutive patients referred to our fracture liaison service from June 2012 to June 2014 after a recent fracture.

**Results:**

Seven hundred nine patients were included, 201 (28 %) with osteoporosis, 391 (55 %) with osteopenia and 117 (17 %) with normal BMD. Mean age was 66.0 ± 9.8 years, 504 (73 %) were women and 390 (57 %) had one or more underlying secondary factor. Evaluation of clinical risk factors using fracture risk assessment tool (FRAX) identified 38 % of patients with ≥1 secondary factor including smoking (18 %), excessive alcohol use (12 %), glucocorticoid use (12 %) and rheumatoid arthritis (3 %). Laboratory investigations revealed chronic kidney disease in 13 %, monoclonal gammopathy also in 13 % and primary or secondary hyperparathyroidism in 1 and 6 %, respectively. Secondary factors for bone fragility were equally prevalent in patients with osteoporosis, osteopenia or normal BMD.

**Conclusions:**

Our findings demonstrate a high prevalence of secondary factors for bone fragility in patients who have sustained a recent fracture, independently of BMD. The significant number of documented factors, which were treatable, suggest that patients who sustained a fracture should be screened for secondary factors for bone fragility regardless of BMD values to optimise secondary fracture prevention.

## Introduction

Osteoporotic fragility fractures are associated with increased morbidity and mortality and growing personal, societal and economic burdens [1–4]. The presence of a fragility fracture has also been shown to significantly increase the risk of future fractures [5, 6]. Over the past decade, fracture liaison services (FLS) have been globally implemented to improve the identification and treatment of patients at risk for a new fracture in a cost-effective approach [7, 8]. Patients who sustain a fracture and who have osteoporosis are offered treatment with bone-modifying agents, but a significant number of patients who sustain a fracture have bone mineral density (BMD) in the osteopenia range [9, 10], and in the Netherlands, these are not generally screened for secondary factors for bone fragility and are not routinely offered anti-osteoporosis treatment [11, 12].

Secondary factors for bone fragility are common in patients with osteoporosis and a fragility fracture [13–15], but data on the prevalence of these factors in patients with osteopenia or normal BMD and a fragility fracture are scarce [13, 15]. Postmenopausal women with osteopenia and fragility fractures have been shown to have poor bone microarchitecture and altered material properties of the bone, which may also be influenced by secondary factors for bone fragility [16–19]. To assess the potential contribution of secondary factors for bone fragility to fracture risk, we set out to evaluate the prevalence of these factors in a cohort of patients who had recently sustained a fracture and who were referred to the FLS for further investigation and management.

## Patients and methods

### Study design

In this prospective cohort study, all patients ≥50 years old with a recent fracture, who were referred to the FLS of the Leiden University Medical Center from June 2012 to June 2014, were screened for secondary factors for bone fragility.

### Patients

**Fig 1:**
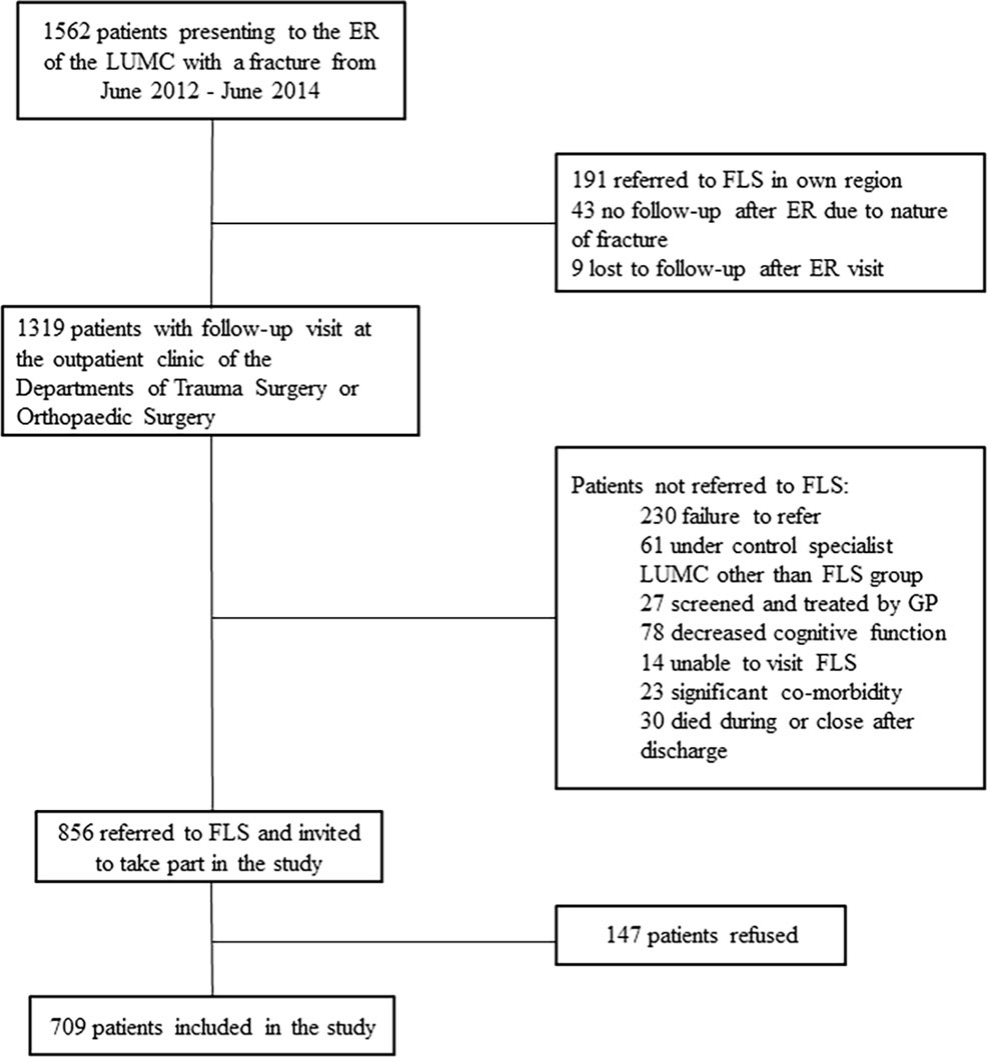
Flowchart of inclusion of patients in the study after presenting to the Emergency Room of the Leiden University Medical Center with a recent fracture. *ER* emergency room, *FLS* fracture liaison service, *GP* general practitioner

Patients were informed of their referral to the FLS during their follow-up visit for primary fracture care at the outpatient clinics of the departments of traumatology and orthopaedic surgery. Excluded from the study were patients with an isolated fracture of the skull, hands or feet, patients with pathological fractures or those with fractures resulting from failure of prosthesis. Patients who had undergone screening for osteoporosis in another hospital were also not included in the study. Patients with impaired cognitive function or poor general condition were also excluded from the study; Fig. 1.

### Study parameters

The following data were collected in all patients: age, gender, height and weight (from which body mass index (BMI) was calculated), a full medical history including detailed fracture history, family history of osteoporosis, dietary calcium intake, age at menopause (early menopause was defined as menopause at age ≤45 years), parental history of hip fracture, alcohol use, corticosteroid use, smoking, current use of medication, including vitamin D supplementation, past or present use of hormone replacement therapy and past or present use of bone-modifying agents. The 10-year probabilities for a major osteoporotic fracture and for a hip fracture were calculated using the World Health Organization (WHO) fracture risk assessment tool (FRAX) algorithm using reference values for the Dutch population [20]. The 10-year fracture probabilities were calculated with and without inclusion of values for femoral neck BMD.

### Bone mineral density

Bone mineral density was measured at the lumbar spine (L1–L4) and at the left and right femoral neck by dual-energy X-ray absorptiometry (DXA) using Hologic QDR Discovery A (Hologic, Bedford, MA, USA). *T* scores were calculated using NHANES III reference values compatible with reference values for the Dutch population. The World Health Organization criteria were used to define osteoporosis, osteopenia and normal BMD. Average values of right and left femoral neck BMD were used in the analysis of data, except when both sides could not be measured due to the presence of a prosthesis.

### Laboratory investigations

Serum was measured for calcium, albumin, inorganic phosphate, alkaline phosphatase, potassium, sodium, ureum, creatinine, TSH, PTH, 25-OH vitamin D and P1NP. Vitamin D deficiency was defined as serum levels of 25-OH vitamin D <50 nmol/L. Renal function was assessed by calculating the estimated glomerular filtration rate (eGFR) using the modification of diet in renal disease (MDRD) formula. Stages of chronic kidney disease were defined as I to V according to the classification of the National Kidney Foundation KDOQI [21]. Primary hyperparathyroidism was diagnosed by hypercalcemia (albumin corrected calcium >2.55 mmol/L) in the presence of an inappropriately normal or elevated PTH level (PTH > 8.0 pmol/L), in the absence of thiazide use. Hyperthyroidism was diagnosed by a repeated TSH level <0.300 mU/L in the presence of a free T4 (fT4) level >22.0 pmol/L and subclinical hyperthyroidism by a TSH level <0.300 mU/L in the presence of a fT4 level between 10.0–24.0 pmol/L, without use of interfering medication. Hypogonadism was screened for in men <70 years of age and was diagnosed by a total testosterone level of <8 nmol/L in a morning sample. Screening for a monoclonal gammopathy using immunofixation was undertaken at the discretion of the treating physician, mostly in case of unexpected osteoporosis and/or multiple fractures, also in the presence of osteopenia. Monoclonal gammopathy of undetermined significance (MGUS) was defined by the presence of M-protein in serum at a concentration of up to 10 g/L, with no signs of organ damage in the form of anaemia, hypercalcemia, kidney insufficiency or bone lesions [22].

### Statistical analysis

All analyses were performed using the SPSS software for Windows (Version 20.0; SPSS Inc., Chicago, IL, USA). Between-group differences in baseline characteristics were assessed using ANOVA, a Chi-square test or a Kruskall Wallis test. The prevalence of secondary factors for bone fragility according to the recently sustained fracture type was assessed using a Chi-square test. Pearson correlation coefficients were used to express correlations between the 10-year FRAX probability calculated with and without femoral neck BMD values (after logarithmic transformation), lumbar spine and mean femoral neck BMD, and the number of secondary factors for bone fragility. Partial correlation coefficients were calculated to assess the correlation between number of secondary factors and lumbar spine and mean femoral neck BMD after adjusting for age, gender and BMI. Multivariate logistic regression analysis was used to assess the contribution of BMD: normal, osteopenia and osteoporosis (variable) to the prevalence of secondary factors for bone fragility (outcome), adjusted for age, gender and BMI.

Differences were considered to be significant at *p* < 0.05.

## Results

In the 2-year study period, 1562 patients presented to the emergency room of the Leiden University Medical Center with a recent fracture. Seven hundred six patients were not referred to the fracture liaison service for a variety of reasons detailed in Fig. 1. Compared to this group, the 856 patients who were referred were younger (68.6 ± 11.2 years vs. 72.2 ± 12.9 years, *p* < 0.001), predominantly female (71 % vs. 69 %) and had sustained less hip fractures (hip fracture/vertebral fracture/non-hip non-vertebral fracture 9/6/85 % vs. 22/8/70 %; *p* < 0.001). There was no significant difference in the number of female patients who were referred to the FLS or not, *p* = 0.229.

Of these 856 patients referred, 709 agreed to be further investigated for the presence of secondary factors for bone fragility and were included in the study. These were 196 men and 513 women, with a mean age of 67.1 ± 10.2 years (range 50.0–94.0 years). Sixty-one (9 %) had a hip fracture, 40 (6 %) a clinical vertebral fracture and 608 (86 %) a non-hip/non-vertebral (NH/NV) fracture.

Two hundred one patients (28 %) had osteoporosis, 391 (55 %) had osteopenia and 117 (17 %) had normal bone mineral density (BMD). Data on FRAX clinical risk factors for fracture and/or laboratory data were incomplete in 23 patients, so that 686 patients were included in the final analysis, 385 of whom had osteopenia and 102 normal BMD.

After stratification of patients according to BMD, there were significant differences in mean age (64.7 ± 9.9 vs. 66.0 ± 9.8 vs. 70.5 ± 10.2 years, *p* < 0.001), gender distribution (34 vs. 29 vs. 17 % male patients, *p* = 0.001) and BMI (28.2 ± 5.1 vs. 26.7 ± 4.1 vs. 24.3 ± 3.8 kg/m^2^, *p* < 0.001) between groups of patients with respectively normal BMD, osteopenia and osteoporosis. There was no difference in biochemical parameters, number of patients with a previous fracture or with a history of parental hip fracture between the three BMD groups; Table 1.

**Table 1:** Characteristics of patients with normal BMD, osteopenia and osteoporosis

	Normal BMD (*n* = 102)	Osteopenia (*n* = 385)	Osteoporosis (*n* = 199)	*p* value
Age (years)	64.7 ± 9.9	66.0 ± 9.8	70.5 ± 10.2	<0.001
Male/female (%)	35/67 (34/66)	113/272 (29/71)	34/165 (17/83)	0.001
BMI (kg/m^2^)	28.2 ± 5.1	26.7 ± 4.1	24.3 ± 3.8	<0.001
Previous facture (%)	41 (40)	163 (42)	83 (42)	0.918
Parental hip fracture (%)	10 (10)	50 (13)	28 (14)	0.560
FRAX score major fracture	5.2 ± 0.6	8.9 ± 0.3	17.0 ± 0.7	<0.001
FRAX score hip fracture	0.5 ± 0.3	2.2 ± 0.2	7.3 ± 0.6	<0.001
Laboratory data:				
Calcium (mmol/L)	2.40 ± 0.12	2.41 ± 0.12	2.41 ± 0.11	0.648
Creatinine (μmol/L)	74.4 ± 15.3	74.7 ± 19.1	72.3 ± 26.4	0.422
PTH (pmol/L)	4.0 ± 2.4	4.4 ± 2.8	4.9 ± 4.2	0.111
25-OH D (nmol/L)	62.7 ± 36.6	57.5 ± 28.2	58.4 ± 30.5	0.677
DXA measurements:				
LS BMD (g/cm^2^)	1.11 ± 0.12	0.95 ± 0.12	0.80 ± 0.14	<0.001
*T* score LS	0.5 ± 1.0	−1.0 ± 1.0	−2.3 ± 1.2	<0.001
FN BMD (g/cm^2^)	0.86 ± 0.10	0.70 ± 0.07	0.58 ± 0.08	<0.001
T score FN	−0.1 ± 0.7	−1.5 ± 0.5	−2.5 ± 0.6	<0.001

*BMI* body mass index, *DXA* dual-energy X-ray absorptiometry, *LS* lumbar spine, *FN* femoral neck, *BMD* bone mineral density

The majority of patients had a non-hip/non-vertebral (NH/NV) fracture [*n* = 586 (85 %)], 60 patients (9 %) had a hip fracture and 40 patients (6 %) had a clinical vertebral fracture. The most prevalent NH/NV fracture was a wrist fracture [*n* = 221 (32 %)] followed by a fracture of the proximal humerus [*n* = 91 (13 %)] and of the ankle [*n* = 79 (12 %)]. Forty patients (67 %) with a hip fracture had a secondary factor for bone fragility, 28 patients (70 %) with a vertebral fracture and 322 patients (55 %) with a NH/NV fracture; *p* = 0.049.

A similar distribution of fractures was observed in patients with normal BMD: 6 (6 %) hip fracture, 4 (4 %) clinical vertebral fracture and 92 (90 %) NH/NV fracture; osteopenia: 33 (9 %) hip fracture, 19 (5 %) vertebral fracture and 333 (86 %) NH/NV fracture; and in patients with osteoporosis: 21 (11 %) hip fracture, 17 (9 %) vertebral fracture and 161 (81 %) NH/NV fracture.

### Secondary factors identified by clinical risk factors using FRAX

In patients with normal BMD, 13 patients (13 %) used ≥3 units of alcohol a day, 11 (11 %) were currently using or had previously used glucocorticoids, 15 (16 %) were active smokers and 3 (3 %) had rheumatoid arthritis; Table 2. Eight patients (9 %) had a history of parental hip fracture, and 41 (40 %) had sustained one or more previous fractures. The median FRAX 10-year fracture probability was 5.2 % for a major osteoporotic fracture and 0.5 % for a hip fracture, and was 7.3 % and 2.2 %, respectively without inclusion of femoral neck BMD in the calculation.

**Table 2:** Prevalence of secondary factors for bone fragility in patients with a recent fracture grouped according to gender and BMD

	Male (*n* = 182)	Female (*n* = 504)	Normal BMD (*n* = 102)	Osteopenia (*n* = 385)	Osteoporosis (*n* = 199)	Total number patients (*n* = 686)
FRAX clinical risk factors:						
Smoking (%)	42 (23)	80 (16)	15 (16)	64 (17)	43 (22)	122 (18)
Use of >3 IU alcohol (%)	30 (17)	49 (10)	13 (13)	44 (11)	22 (11)	79 (12)
Glucocorticoids (%)	14 (8)	66 (13)	11 (11)	42 (11)	27 (14)	80 (12)
Rheumatoid arthritis (%)	2 (1)	20 (4)	3 (3)	11 (3)	8 (4)	22 (3)
Early menopause (%)	–	96 (19)	13 (13)	45 (12)	38 (19)	96 (14)
Laboratory-based factors						
Chronic kidney disease (%)	15 (8)	77 (15)	10 (10)	53 (14)	29 (15)	92 (13)
MGUS (%)	25 (14)	65 (13)	5 (5)	46 (12)	39 (20)	90 (13)
1° hyperparathyroidism (%)	–	7 (1)	1 (1)	4 (1)	2 (1)	7 (1)
2° hyperparathyroidism (%)	7 (4)	35 (7)	3 (3)	25 (7)	14 (7)	42 (6)
Hyperthyroidism (%)	1 (1)	12 (2)	1 (1)	7 (2)	5 (3)	13 (2)
Hypogonadism (%)	8 (4)	–	1 (1)	6 (2)	1 (1)	8 (1)
Patients with >1 factor (%)	93 (51)	297 (59)	48 (47)	219 (57)	123 (62)	390 (57)

*IU* international unit

In patients with osteopenia, 44 patients (11 %) used more than 3 units of alcohol per day, 42 (11 %) were currently using or had previously used glucocorticoids, 64 (17 %) were active smokers and 11 (3 %) had rheumatoid arthritis; Table 2. Fifty patients (13 %) had at least one parent with a history of a hip fracture, and 163 patients (42 %) had sustained a previous fracture.

The median 10-year fracture probability as calculated by FRAX was 8.9 % for a major osteoporotic fracture and 2.2 % for a hip fracture. Without inclusion of femoral neck BMD in the calculation, the median 10-year fracture probability was 9.4 % for a major osteoporotic fracture and 2.8 % for a hip fracture.

In patients with osteoporosis, 22 patients (11 %) used ≥3 units of alcohol a day, 27 (14 %) were currently using or had previously used glucocorticoids, 43 (22 %) were active smokers and 8 (4 %) had rheumatoid arthritis; Table 2. Twenty-eight patients (14 %) had a history of parental hip fracture, and 83 (42 %) had sustained one or more previous fractures.

The median 10-year fracture probability with inclusion of femoral neck BMD was 17 % for a major osteoporotic fracture and 7.3 % for a hip fracture, and respectively 16 and 6.2 % without inclusion of femoral neck BMD in the calculation.

**Fig. 2:**
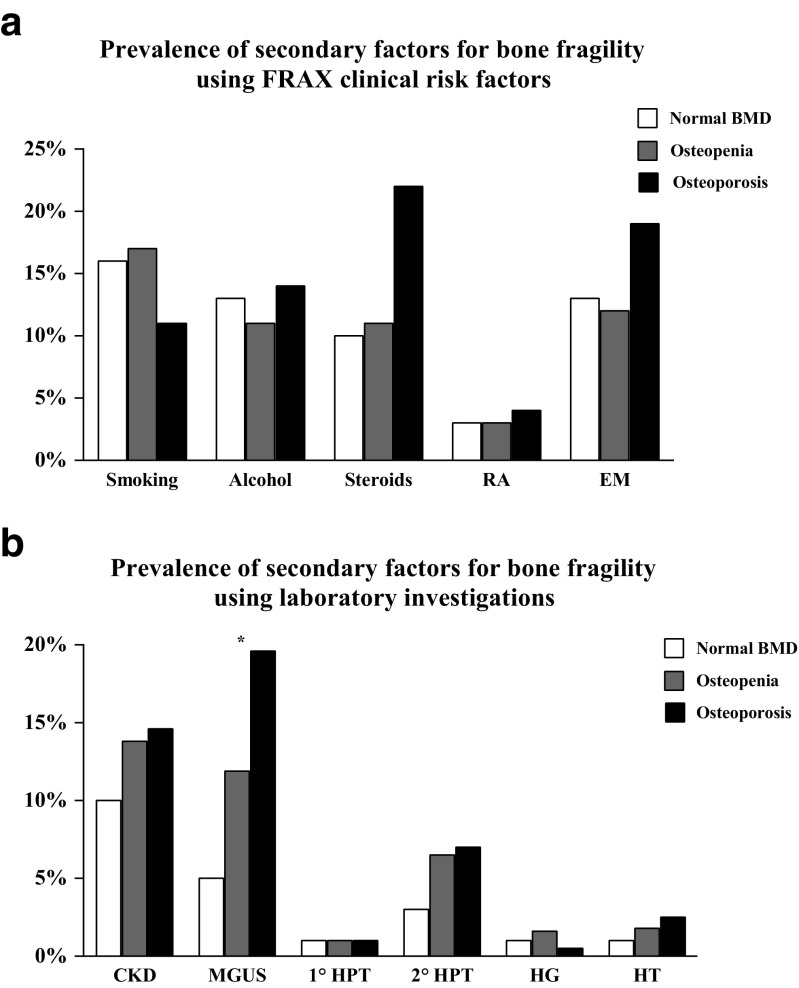
**a** No significant difference in the prevalence of underlying secondary factors for bone fragility between patients with normal BMD (*white bars*) osteopenia (*grey bars*) or osteoporosis (*black bars*) using FRAX. *RA* rheumatoid arthritis, *EM* early menopause. **b** Prevalence of underlying secondary factors for bone fragility by laboratory investigations in patients with normal BMD (*white bars*), osteopenia (*grey bars*), and osteoporosis (*black bars*). Significantly different prevalence of MGUS in patients between the groups. *CKD* chronic kidney disease, *MGUS* monoclonal gammopathy of undetermined significance, *1° HPT* primary hyperparathyroidism, *2° HPT* secondary hyperparathyroidism, *HG* hypogonadism, *HT* hyperthyroidism. *p = 0.001

There was a significant difference in the 10-year FRAX probability for fracture between patients with normal BMD, osteopenia or osteoporosis, with or without inclusion of femoral neck BMD measurements; Table 1. A similar number of patients with normal BMD, osteopenia or osteoporosis had an underlying factor for bone fragility as identified by clinical risk factors for fracture using FRAX (41 patients with normal BMD (40 %) vs. 164 patients with osteopenia (43 %) vs. 95 patients with osteoporosis (48 %); *p* = 0.381). There was no difference in the prevalence of any individual clinical risk factor studied between the groups; Fig. 2a.

There was a significant difference in the 10-year FRAX probability for fracture calculated with BMD values between patients with ≥1 secondary factor and patients without (12.0 vs. 8.5 %; *p* < 0.001), and in the 10-year FRAX probability without BMD values (13.0 vs. 8.3 %; *p* < 0.001). Interestingly, there was a significant relationship between the cumulative number of factors for bone fragility and the 10-year FRAX probability (*r* = 0.336, *p* < 0.001), and in the 10-year FRAX probability calculated without BMD values (*r* = 0.359, *p* < 0.001). After stratification for BMD status, this relationship remained for normal BMD (*r* = 0.286, *p* = 0.004 for FRAX with BMD and *r* = 0.456, *p* < 0.001 for FRAX without BMD), osteopenia (*r* = 0.313, *p* < 0.001 for FRAX with BMD and *r* = 0.311, *p* < 0.001 for FRAX without BMD) and osteoporosis (*r* = 0.361, *p* < 0.001 for FRAX with BMD and *r* = 0.346, *p* < 0.001 for FRAX without BMD).

### Secondary factors identified by laboratory investigations

Laboratory investigations identified an underlying factor for bone fragility in 18 (18 %) of patients with normal BMD, in 112 (29 %) of patients with osteopenia and in 69 (35 %) of patients with osteoporosis.

In patients with normal BMD, chronic kidney disease was diagnosed in ten patients (10 %), all of whom had chronic kidney disease (CKD) stage III. One patient (1 %) had primary hyperparathyroidism, and three (3 %) had secondary hyperparathyroidism (associated with low 25-OH vitamin D levels in two, and combined vitamin D deficiency and renal failure in one). One patient (1 %) had hyperthyroidism, one male patient had hypogonadism and five (5 %) patients were diagnosed with MGUS.

In patients with osteopenia, chronic kidney disease was diagnosed in 53 patients (14 %), of whom 49 had stage III CKD and 4 had stage IV CKD. Four patients (1 %) had primary hyperparathyroidism and 25 (7 %) had secondary hyperparathyroidism (associated with low 25-OH vitamin D levels in 15, and combined vitamin D deficiency and renal failure in 8). Seven patients (2 %) had hyperthyroidism (5 subclinical), 6 men had hypogonadism and 46 (12 %) patients were diagnosed with MGUS.

In patients with osteoporosis, chronic kidney disease was diagnosed in 29 patients (15 %), of whom 26 had stage III CKD and 3 had stage IV CKD. Two patients (1 %) had primary hyperparathyroidism, and 14 had secondary hyperparathyroidism (associated with low 25-OH vitamin D levels in 7, and combined vitamin D deficiency and renal failure in 7). Five patients (3 %) had hyperthyroidism (2 subclinical), 1 male patient had hypogonadism and 39 (20 %) patients were diagnosed with MGUS.

All underlying factors for bone fragility identified by laboratory investigations were equally prevalent in patients with normal BMD, osteopenia or osteoporosis, except for MGUS (5 vs. 12 vs. 20 %; *p* = 0.001); Fig. 2b. One patient with osteoporosis was diagnosed as having multiple myeloma.

The odds of a patient with osteopenia having an MGUS were 2.71 times higher than those of a patient with normal BMD, and the odds of a patient with osteoporosis having an MGUS were 4.81 times higher than those of a patient with normal BMD. There was no association between BMD and the odds for any other factor for bone fragility; Table 3.

**Table 3:** Odds ratio for factors for increased bone fragility in patients with osteoporosis and osteopenia, compared with patients with normal BMD

	Odds ratio [95 % CI]	*p* value
FRAX:		
Smoking	1.610 [0.795–3.259] 1.086 [0.578–2.038]	0.186 0.798
Use of >3 IU alcohol	0.916 [0.413–2.033] 0.858 [0.436–1.689]	0.829 0.657
Glucocorticoids	1.454 [0.645–3.273] 1.089 [0.530–2.238]	0.367 0.817
Rheumatoid arthritis	1.135 [0.263–4.899] 0.952 [0.252–3.605]	0.865 0.942
Early menopause	1.660 [0.762–3.613] 0.958 [0.470–1.952]	0.202 0.905
Laboratory investigations:		
Chronic kidney disease	1.196 [0.510–2.805] 1.532 [0.713–3.290]	0.681 0.274
MGUS	4.805 [1.745–13.231] 2.713 [1.037–7.098]	0.002 0.042
1° hyperparathyroidism	2.580 [0.156–42.571] 1.808 [0.170–19.278]	0.508 0.624
2° hyperparathyroidism	2.212 [0.574–8.518] 2.470 [0.708–8.613]	0.249 0.156
Hypogonadism	1.330 [0.068–25.847] 2.135 [0.239–19.096]	0.851 0.498
Hyperthyroidism	1.883 [0.194–18.316] 1.822 [0.213–15.609]	0.586 0.584

For every risk factor listed, odds ratio refers to patients with osteoporosis at the top line and to patients with osteopenia at the second line. Patients with normal BMD are used as reference

*IU* internal unit, *MGUS* monoclonal gammopathy of undetermined significance

### Relationship between number of secondary factors for bone fragility and BMD measurements

Three hundred ninety patients (57 %) had one or more underlying secondary factor for bone fragility. The majority of patients (*n* = 205 [30 %]) had one underlying factor, 122 patients (18 %) had two and 63 (9 %) had three or more factors; 38 % of all observed underlying factors were identified by laboratory investigations, and 41 % of all factors were reversible. There was an inverse relationship between the number of underlying factors for bone fragility per patient and mean femoral neck BMD values (*r* = −0.215, *p* < 0.001), which persisted after adjusting for age, gender and BMI (*r* = −0.192, *p* < 0.001).

In patients with normal BMD, 29 patients (28 %) had one underlying factor, 13 patients (13 %) had two factors and 6 (6 %) had three or more factors. In patients with osteopenia, the majority of patients (*n* = 122 [32 %]) had one underlying factor, 69 patients (18 %) had two and 28 (7 %) had three or more factors. In patients with osteoporosis, 54 patients (27 %) had one secondary factor for bone fragility, 40 patients (20 %) had two factors and 29 (15 %) had three or more factors.

### Vitamin D deficiency

Vitamin D deficiency was prevalent in 292 (43 %) of the 686 patients, of whom 97 had serum vitamin D levels <25 nmol/L. Vitamin D deficiency was documented in 43 patients (42 %) with normal BMD, in 166 patients (43 %) with osteopenia and in 83 patients (42 %) patients with osteoporosis.

## Discussion

Our data from this study demonstrate that 57 % of patients, who recently sustained a fracture and who were subsequently referred to our fracture liaison service, had one or more underlying secondary factor for bone fragility. Interestingly, these secondary factors were equally prevalent in our cohort of patients with a recent fracture whether they had normal bone mineral density (BMD), osteopenia or osteoporosis (47 vs. 57 vs. 62 % respectively; *p* = 0.05). Our data also show a significant inverse relationship between the number of underlying factors for bone fragility and femoral neck BMD. The most prevalent underlying factors for bone fragility were smoking (18 %), chronic kidney disease (13 %) and MGUS (13 %). Of clinical relevance is that 41 % of all documented secondary factors for bone fragility were reversible (hyperthyroidism, primary and secondary hyperparathyroidism, hypogonadism in men or potentially reversible such as excessive alcohol use and smoking). This may hold significant clinical implications in the management of these patients as reversal of these factors may be associated with an improvement in bone strength, thus contributing in time to a decrease in bone fragility. In addition, 43 % had serum levels of 25-OH vitamin D <50 nmol/L.

The prevalence of secondary factors for bone fragility has been previously reported in patients with a recent fracture [13, 15]. Although the majority of previously published data studied the prevalence of underlying factors for bone fragility in patients with osteoporosis [13–15, 23–27], our findings suggest a similar prevalence of these factors regardless of BMD measurements after a recent fracture. A novel approach we pursued in this analysis was to compare the prevalence of underlying factors for bone fragility between the groups of patients with normal BMD, osteopenia or with osteoporosis in our cohort of 686 patients aged ≥50 years who had sustained a recent fracture.

As expected, the FRAX 10-year probability for a fracture was significantly different in patients with normal BMD, osteopenia or osteoporosis. This was also the case when femoral neck BMD was not used in the calculation of the FRAX. Our data also demonstrate that patients with one or more secondary factor for bone fragility had a higher 10-year FRAX fracture probability compared to patients with no documented secondary factor(s). Interestingly, there was a positive relationship between the cumulative number of secondary factors for bone fragility and FRAX independently of BMD status, which suggests that FRAX may not fully capture the contribution of the cumulative effect of these factors on fracture risk.

Our study has strengths as well as limitations. One of its main strengths is that all fracture patients were identified at source at the outpatient clinics of the departments of traumatology and orthopaedic surgery by a dedicated fracture nurse from our FLS resulting in the successful referral of >50 % patients to our FLS for screening for osteoporosis. Over the last decade, there has been an increasing drive to develop and implement FLSs on an international scale for the secondary prevention of fractures [28]. These FLSs ensure that future fracture risk is assessed in all patients who have sustained a recent fracture, including the risk of falling [29]. These FLSs also secure that treatment with bone-modifying agents is initiated if and when required. Previous studies demonstrated that the implementation of an FLS was cost-effective and effectively reduced mortality and the incidence of subsequent non-vertebral fractures [8, 30].

A further strength of the study is the availability of data on clinical risk factors using FRAX, of BMD data and of laboratory data on the most common secondary factors for bone fragility. A main limitation of the study is that due to regional hospital policy, the majority of patients who presented to our emergency room with a hip fracture were transferred to other hospitals in the region for primary fracture care, which could have influenced patient characteristics, especially age. The limited screening for monoclonal gammopathy of undetermined significance (MGUS) may also be considered as a limitation of our study, as data may have been confounded by the bias of the treating physician’s decision to screen patients at higher risk for underlying secondary factors for bone fragility. However, this would have led to an underestimate rather than an overestimate of the prevalence of an MGUS. LS BMD measurements were not adjusted for the presence of degenerative changes, which might explain the absence of a relationship between the number of underlying factors for bone fragility and LS BMD. However, we also found no correlation between the number of underlying factors and LS BMD in patients stratified by age groups (data not shown).

Our data, demonstrating a high prevalence of secondary factors for bone fragility, independently of BMD status, in a cohort of patients who had sustained a recent fracture, hold significant clinical implications in the management of these patients, as nearly half of these factors were potentially reversible. Our findings suggest that screening for underlying secondary factors for bone fragility should be considered in the setting of fracture liaison services, not only in patients with osteoporosis but also in those with osteopenia or normal BMD. Whether reversing secondary factors for bone fragility would result in more optimal secondary prevention of fractures remains to be established by long-term studies.
